# The effects of radiofrequency on cartilage: A systematic review of preclinical evidence in animals and humans

**DOI:** 10.1002/jeo2.70297

**Published:** 2025-06-01

**Authors:** Umile Giuseppe Longo, Daniela Lo Presti, Youssef Raffoul Arab Orozco, Dario Neyjat, Sergio De Salvatore, Francesca De Tommasi, Margaux D'Hooghe, Alessandro de Sire, Kristian Samuelsson

**Affiliations:** ^1^ Fondazione Policlinico Universitario Campus Bio‐Medico Roma Italy; ^2^ Department of Medicine and Surgery, Research Unit of Orthopaedic and Trauma Surgery Università Campus Bio‐Medico di Roma; ^3^ IRCCS Ospedale Pediatrico Bambino Gesù Rome Italy; ^4^ Department of Medicine University of Navarra Pamplona Spain; ^5^ Department of Medical and Surgical Sciences University of Catanzaro “Magna Grecia” Catanzaro Italy; ^6^ Department of Orthopaedics, Institute of Clinical Sciences, The Sahlgrenska Academy University of Gothenburg Gothenburg Sweden

**Keywords:** ablation, bipolar, cartilage, monopolar, radiofrequency

## Abstract

**Purpose:**

Radiofrequency works by applying continuous waveforms which ablate the surface of the cartilage. Despite its utility, the limits and possible risks of this technology have not been thoroughly investigated in the literature. This review aims to systematically summarise the most recent studies regarding the effects of the various radiofrequency on cartilage and compare the side effects they may bring.

**Methods:**

A search following the PRISMA guidelines was conducted on scientific databases: Web of Science, Embase CINAHL, ProQuest and Medline from May 2021 to January 2025. The databases were searched from their inception to September 2023. Only articles in English were included. In vivo, ex vivo, cadaveric, and in vitro studies were all included. To assess the risk of bias, Rob2 and ROBINS‐I were used.

**Results:**

Twenty‐eight studies were included in this systematic review. The level of evidence of the included studies ranged from level II to III. The most analysed specimen was human tissue, followed by swine, ovine, bovine, equine, canine and rabbit. Cartilage damage was reported in most of the articles. Monopolar radiofrequency was considered safer than bipolar radiofrequency. However, controversial results were found on the superiority of radiofrequency energy to mechanical debridement.

**Conclusion:**

This review shows that there is not enough evidence to precisely identify the possible dangers or benefits that may come from the use of radiofrequency energy on cartilage. Therefore, the real risks of radiofrequency are not fully explored. Further research needs to include radiofrequency energy in the clinical and surgical environment.

**Level of Evidence:**

Level II, evidence from one or more well designs randomised control trials (RCT).

**Abbreviations**
BRF: bipolar radiofrequency
ICRS: International Cartilage Repair Society Score
MD: mechanical debridement
MRF: monopolar radiofrequency
NRCT: non‐randomised controlled trial
OARSI: Osteoarthritis Research Society International (OARSI) Score
RCT: randomised control trial
RF: radiofrequency
RFE: radiofrequency energy
Rob2: risk of bias 2
ROBINS‐I: risk of bias in non‐randomised studies

## INTRODUCTION

Introduced at the beginning of the 20th century, the application of radio frequencies (RF) in the surgical environment has been widespread since the appearance of the Bovie RF generator in 1926 [40]. RF have a broad utility spectrum in modern societies, with applications in several fields [17]. The cutting with RFs is performed by emission of continuous radio frequency waveforms, which ablate soft tissue leaving a collateral damage zone. Two basic RF energy systems with controlled probes for clinical application (monopolar and bipolar) can accomplish this process. RF uses a mechanism based on oscillating electrical current forcing collisions between charged molecules and ions, transforming into heat [2, 4]. Ablation refers to the complete removal of tissue using thermal energy, often leading to collateral damage to surrounding structures. In the context of RF, ablation occurs when high‐frequency alternating currents generate heat sufficient to vaporise tissue [2, 4]. Coblation as a surgical technique involves the administration of RF in a much more retractable manner. It prevents the removal of underlying healthy cartilage without decreasing its efficacy in eliminating the targeted cells by creating a low‐temperature plasma field [1, 21]. This heats the tissue to a temperature of 60°C–70°C, a temperature that is vastly inferior to standard cautery, thus reducing tissue damage [5, 39]. This technique has been widely used in orthopaedic surgery to treat cartilage defects.

The three significant factors for the attractiveness of RF are the rapid smoothing and contouring of fibrillated cartilage without leaving irregularities, low complication rate, and low chondrocyte injury or necrosis near the regions that were exposed [12, 26, 36] in contrast to motorised shavers, which have been commonly used to remove denatured and dissociated cartilage, even though no consensus has been reached.

However, some studies investigated RF's safety by evaluating the chondral cell viability and other possible adverse effects [52]. Multiple studies have reported that the excessive temperature reached by the probes of bipolar radiofrequency (BRF) and monopolar radiofrequency (MRF) may lead to cell death due to extreme thermal damage [6, 7, 11, 12, 13, 25, 31, 33] to the tissue surrounding the lesion. Compared to the clinical benefit of smoother, less fibrillated cartilage achieved with RF, this leads to unacceptable thermal damage. There is an important distinction as the two modalities of delivering RF, namely MRF and BRF, appear to have side effects of different magnitude depending on whether MRF or BRF provided treatment. The former seems to be associated with fewer side effects, such as thermal damage and chondrocyte death, as reported by Cook et al. [6] and Edwards et al. [10] meanwhile, the latter, as written by Lu et al. [30] who brings forward data demonstrating that the penetration caused by bipolar RF extends as far as 92% of the total depth cartilage. As previously stated, RF carries a considerable number of risks.

Moreover, according to several authors, also other variables such as lavage solution temperature [5, 32], pressure [30, 35] and time of application could influence the damage in treated tissues [32, 41].

This systematic review aims to evaluate the preclinical evidence on the effects of RF on cartilage, analysing both its potential benefits and associated risks, and to compare its outcomes with alternative cartilage treatment modalities.

## METHODS

### Study selection

The research question was defined by using a PICO approach: Population (P); Intervention (I); Comparison (C); Outcome (O). The objective of this systematic review was to report the most recent findings about the effects (O) of the use of several types of specimen (P) treated by RF (I) compared to mechanical debridement (MD) or untreated cartilage.

### Eligibility criteria

Only articles written in English were included. Articles that mentioned: RF, both monopolar or bipolar, and damage to cartilage caused by RF were included. Only studies specifying the joint from which the cartilage was obtained were included. Studies that vaguely referred to “articular cartilage” without indicating a specific joint (e.g., knee, hip and shoulder) were excluded, as anatomical location is a critical factor in cartilage response to RF energy.

The study designs included are randomised control trials, non‐randomised control trials, and controlled laboratory studies. Animal, in vitro, cadaveric and ex vivo studies were all included. Articles that mentioned cartilage and/or bone cancer were excluded. Other surgical techniques were excluded. Technical notes, letters to editors and other grey literature (not indexed in peer reviewed journals) were also discarded. Studies that did not specify whether the applied RF was MRF or BRF were included in this review, provided that they contained relevant outcome data on cartilage damage. The objective of this study was to assess the overall effects of RFE on cartilage, rather than to perform a direct monopolar versus bipolar RF comparison. Future studies should focus exclusively on either monopolar or bipolar RF to allow for a more specific comparative analysis.

### Information sources, search strategy

**Figure 1:**
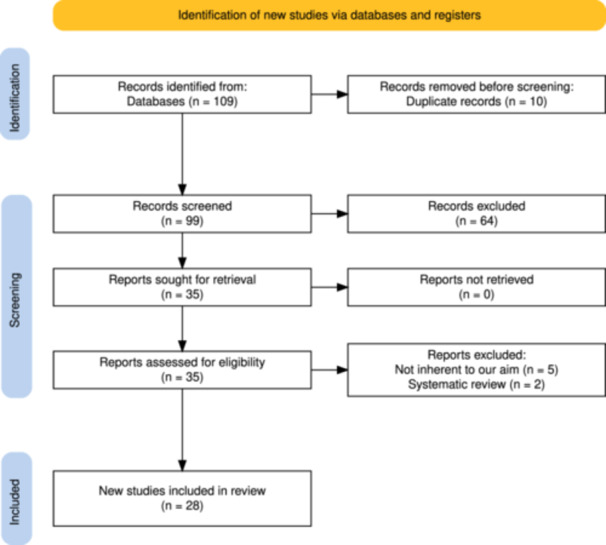
Prisma flowchart.

The following databases were used to identify the studies: Web of Science, Embase CINAHL, ProQuest, Medline and Scopus. The search was performed between June 2021 and January 2025. All the databases were screened from the inception to September 2023. The search followed PRISMA guidelines for systematic reviews (Figure 1).

The databases were searched with the hereinafter search string: (“radiofrequencies” OR “radiofrequency” OR “coblation” OR “coblator” OR “radiofrequent”[All Fields]) AND (“effect”[All Fields] OR “effecting”[All Fields] OR “effective”[All Fields] OR “effectively”[All Fields] OR “effectiveness”[All Fields] OR “effectivenesses”[All Fields] OR “effectives”[All Fields] OR “effectivities”[All Fields] OR “effectivity”[All Fields] OR “effects”[All Fields] OR (“damage”[All Fields] OR “damaged”[All Fields] OR “damages”[All Fields] OR “damaging”[All Fields])) AND (“cartilage”[All Fields] OR “cartilage”[All Fields] OR “cartilages”[All Fields] OR “cartilages”[All Fields] OR “cartilageous”[All Fields]) AND (“in vivo” OR (“in”[All Fields] AND “vivo”[All Fields]) OR “in vivo”[All Fields] OR (“ex”[All Fields] AND “vivo”[All Fields]) OR (“laboratorial”[All Fields] OR “laboratories”[All Fields] OR “laboratories”[All Fields] OR “laboratory”[All Fields] OR “laboratory s”[All Fields])).

### Selection process

The articles were screened by two authors (YA and DN). The two worked independently using CADIMA [20] to screen the title and abstract first, and then full text of the included studies. As to disagreements, they were sorted out between them until a consensus was reached. At first, only the titles were read, then the abstract of the articles were read. Finally, the remaining articles were read in full text to ascertain whether they could be included. In case of disagreement, a third author (SDS) assessed the analysis of the article.

### Data items

The data items extracted from the studies were: author, date of publication, type of RF, sample size, type of animal and type of cartilage subjected to the treatment. The outcome items extracted was ‘Cartilage Damage’ and ‘Damage induced by radio frequency’.

### Study risk of bias assessment

**Figure 2:**
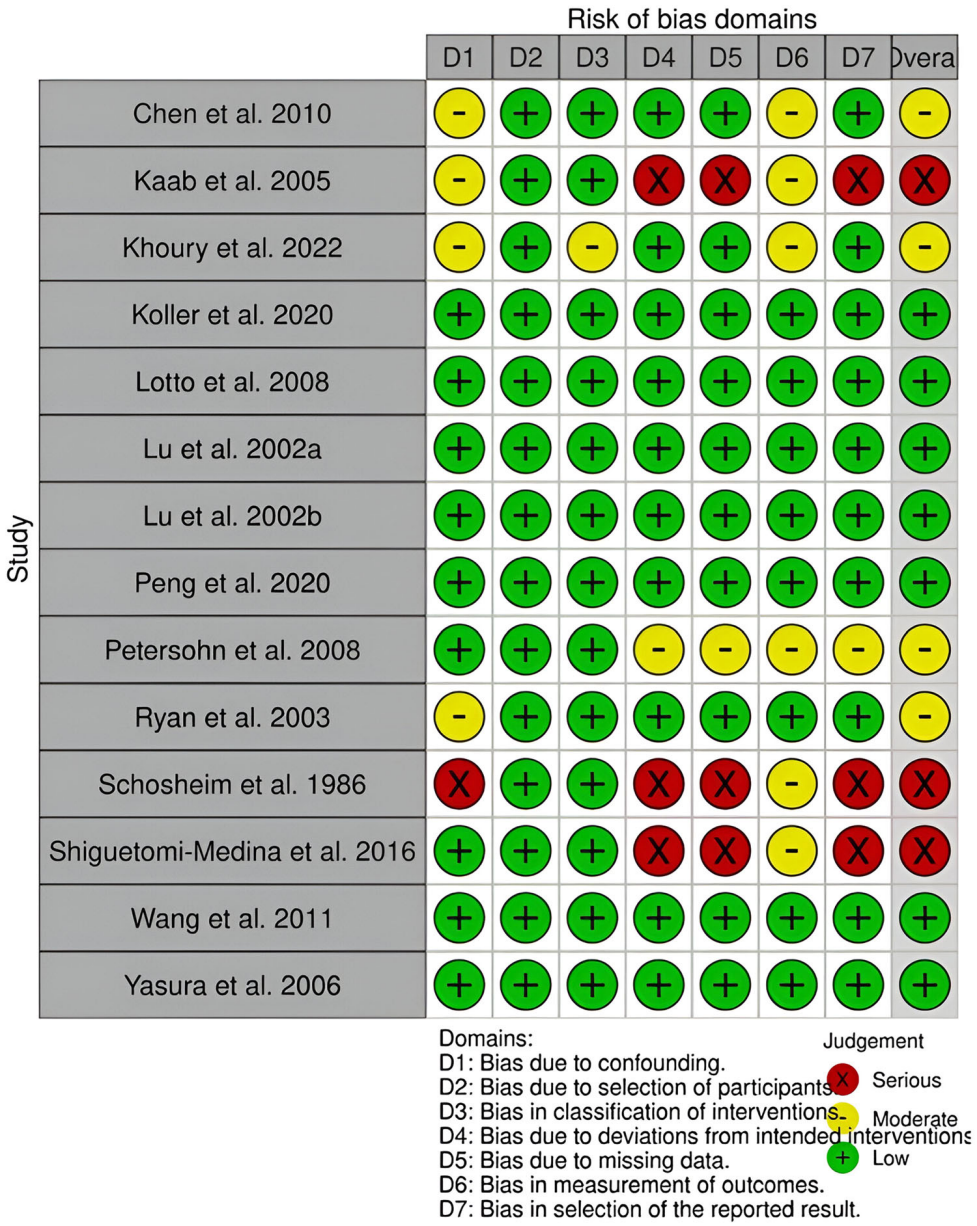
Robins tool for assessment of biases.

**Figure 3:**
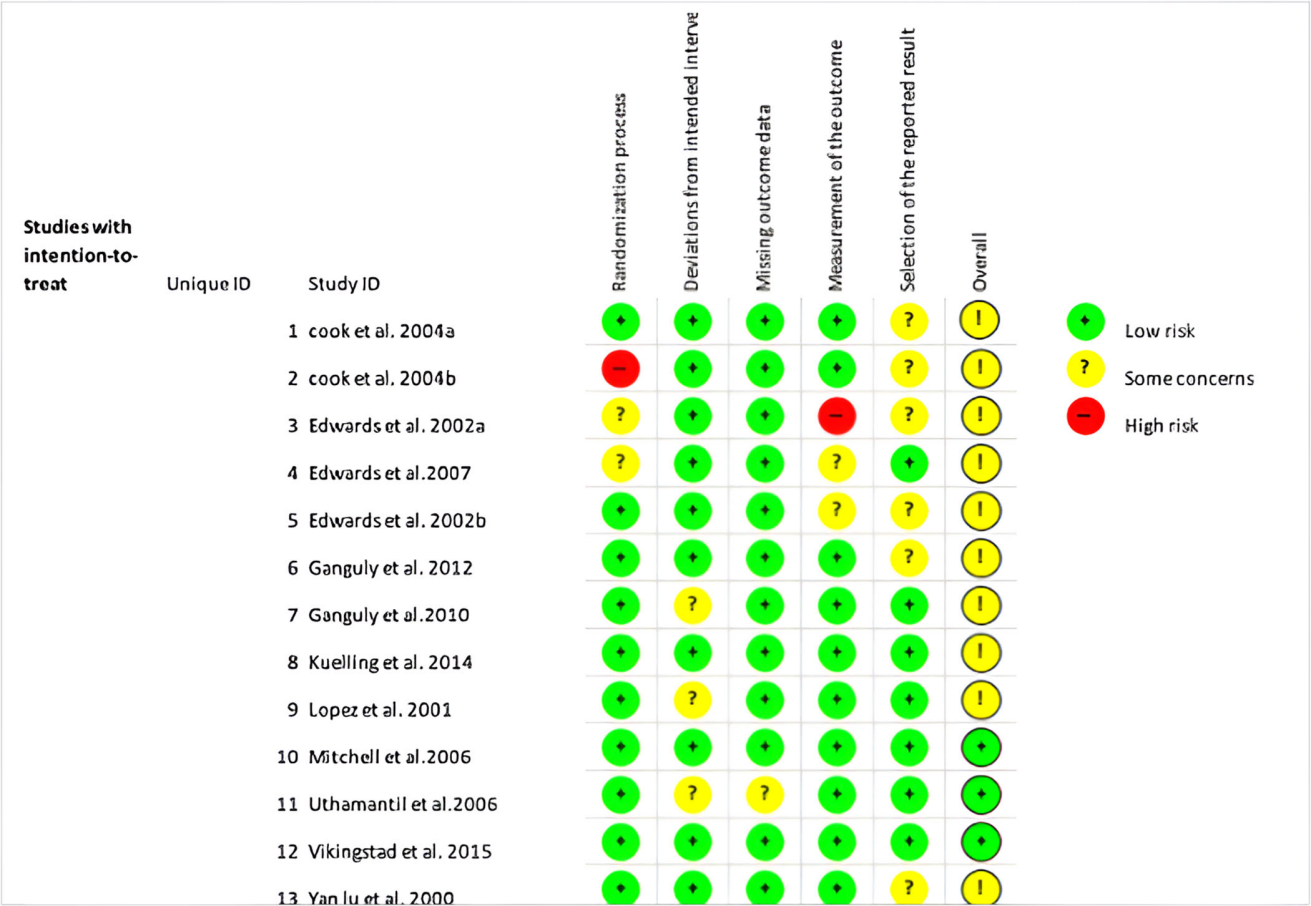
Rob2 tool for assessment of biases.

To check for bias in the selected studies ROBINS‐I [46] (Figure 2) for non‐randomised trials and RoB2 [47] (Figure 3) for randomised trials were used. The articles were independently analysed using these tools by the two authors (YA and DN).

## RESULTS

### Study selection

One hundred and seven studies were found through the search, and grey literature and unpublished articles were not considered. Sixty‐seven were omitted during the title and abstract examination, while four [8, 9, 29, 40] were removed through full‐text analysis as described above. Of these, 28 studies were included in this systematic review. A Prisma flowchart of the proceedings is reported in Figure 1.

### Study characteristics

The included study types were: 13 NRCTs [5, 15, 21, 23, 28, 31, 32, 41, 42, 44, 45, 52, 54], 12 RCTs [6, 7, 10, 12, 13, 14, 22, 27, 33, 35, 49, 51] and three controlled laboratory studies [11, 19, 43]. The level of evidence of the included studies ranged from level II [6, 7, 10, 11, 12, 13, 14, 22, 27, 33, 35, 49, 51] to III [5, 15, 19, 21, 23, 28, 30, 32, 41, 42, 43, 44, 45, 52, 54].

Among the specimens studied: The majority of studies were carried out on human tissue nine [6, 11, 13, 14, 21, 28, 31, 33, 54], followed by swine [42, 45, 51, 52], ovine [15, 23, 27, 33] and bovine [11, 19, 35, 41] found in four articles, and equine [12, 43, 49] found in three articles, canine [7] and rabbit [44] in one.

All the study characteristics are summarised in Table 1.

**Table 1:** Study characteristics and conclusions.

Author	Year	Type of study	Level of evidence	Sample size	Type of animal	Type of (RF) radio frequency	Type of cartilage	Conclusions
Chen et al.	2010	NRCT	III	352	Human	Coblation	Temporomandibular joint	Coblation was found to be significantly more effective than MD, MRF, and BRF in anterior release, adhesion ablation, chondroplasty, and discoplasty, with minimal or no damage to the surrounding cartilage
Cook et al.	2004	RCT	II	72	Canine	Bipolar RF	Articular cartilage	Bipolar RF caused histologic damage and reduced permeability, potentially leading to long‐term detrimental effects
Cook et al.	2004	RCT	II	240	Human	Monopolar, bipolar RF	Aricular cartilage knee	Both monopolar and bipolar RF caused cartilage damage, but MRF treatment resulted in biomechanically more viable cartilage compared to BRF
Edwards et al.	2002	RCT	II	15	Bovine	Monopolar and bipolar RF	Osteochondral sections from the femoropatellar joint.	BRF causes deeper cell death due to higher matrix heating, with fluid flow further increasing surrounding cartilage temperature
Edwards et al.	2007	RCT	II	16	Equine	Non‐specified RF	Patellar	RFE caused greater chondrocyte death and morphological loss compared to mechanically debrided cartilage and controls
Edwards et al.	2002	RCT	II	22	Human	Bipolar RF, monopolar RF	Osteochondral section knee	The study found deeper chondrocyte death with BRF, affecting the subchondral bone in 13 out of 20 cases, while MRF caused no subchondral damage
Ganguly et al.	2012	RCT	II	???	Human	Non‐ablation RF	Unspecified (ostechondral specimens total knee replacement)	No necrotic tissue was found after 1 or 96 hours, and differentiated cell function remained intact
Ganguly et al.	2010	RCT	II	24	Human	Monopolar, bipolar and non‐ablation RF	Articular cartilage of the knee joint	Ablation systems cause tissue necrosis and collateral damage, while non‐ablation systems preserve cartilage integrity
Grana et al.	2006	NRCT	III	136	sheep	Different types	Menisci from hind limbs	RF treated tissue failed at a higher load compared to the mechanically treated one
Kääb et al.	2005	NRCT	III	18	Sheep	Monopolar RF	Femoral condyle cartilage	Detrimental effects on cartilage were seen 24 weeks after the treatment
Khoury et al.	2022	NRCT	III	2	Bovine	Bipolar RF	patellar, condylar, and trochlear	AB3 showed a shallower penetration depth compared to AB4
Koller et al.	2020	NRCT	III	6	Human	Non‐specified RF	Patellar	RF may be unsuitable for Grade 2 patellar cartilage defects, as T2 values normalise initially but exceed preoperative levels by the 4th and 12th month
Kuelling et al.	2014	RCT	II	23	Rabbits	Coblation	Intervertebral discs	Coblation RF effectively treated stabbed intervertebral discs, promoting pro‐resolving molecule activation and preserving disc architecture
Lopez et al.	2001	RCT	II	24	Ovine	Monopolar RF	Meniscal	Thermal damage was confined to the treatment site, with no histological differences between the 10 W and 15 W groups, though the affected area tended to be larger in the 10 W group
Lotto et al.	2008	NRCT	III	48	Human	Monopolar RF	Articular cartilage of the knee joint	mRFE was more effective than a motorised shaver in achieving a smooth surface and reducing total tissue impact
Lu et al.	2000	RCT	II	36	Sheep	Monopolar RF	Articular cartilage	Monopolar radiofrequency caused long‐term cartilage damage in this study
Lu et al.	2002	NRCT	III	16	Human	Monopolar RF	Osteochondral section Knee	A 37°C lavage solution enables the wand to reach its preset temperature faster, reducing chondrocyte death compared to a 22°C solution
Lu et al.	2002	NRCT	III	42	Human	Monopolar and bipolar RF	Osteochondral section Knee	RF‐induced damage is time‐dependent for both monopolar and bipolar RF, with increasing exposure time leading to deeper chondrocyte death. Damage severity varied across different exposure durations for both modalities
Mitchell et al.	2006	RCT	II	13	Bovine	Monopolar RF	Articular cartilage	Lower power settings reduce tissue damage, with the best application technique being a rapid pass with minimal pressure
Peng et al.	2020	NRCT	III	6	Bovine	Bipolar RF	Articular cartilage on femoral condyle	RF effectively smooths and contours cartilage, but its damage is dose‐dependent and increases with longer treatment duration
Petersohn et al.	2008	NRCT	III	2	Porcine	Bipolar RF	Lumbar disks	Cells in the nucleus polposus exhibited damage like loss of celluar detail and coarseness of the fibrillar matrix
Ryan et al.	2003	NRCT	III	22	Equine	Bipolar RF	Patellar	Increasing power leads to greater cell viability loss and proteoglycan degradation in a dose‐dependent manner
Schosheim et al.	1986	NRCT	III	16	Rabbits	Bipolar RF	Meniscal	The study found minimal RF‐induced thermal damage but noted increased heating due to the absence of water. Histological changes included tissue hypercellularity and oedema three months post‐resection
Shiguetomi‐Medina et al.	2016	NRCT	III	40	Swine	Non‐specified RF	Tibial Cartilage	The study reports damage to the articular cartilage with the formation of bone bridges and the disruption of physeal morpholgy
Uthamanthil et al.	2006	RCT	II	30	Equine	Monopolar and bipolar RF	Patellar	All treated cartilages showed a reduced aggregate modulus. The mRF‐treated group had the highest stiffness among treatments (bRF, MD, and control) and maintained a higher aggregate modulus than MD
Vikingstad et al.	2015	RCT	II	17	Swine	Non‐specified RF	Achilles tendon, patella	In the short term, RF ablation caused tendon damage, including longitudinal shortening, superficial hardening, and wrinkling. Biomechanically, rupture occurred earlier than in controls, and histologically, collagen fibre architecture was disrupted
Wang et al.	2012	NRCT	III	12	Porcine	Bipolar RF	Articular cartilage of the knee joint	For bipolar RF, ‘ablation mode’ at higher power reduces thermal radiation injury, whereas ‘coagulation mode’ increases it
Yasura et al.	2006	NRCT	III	86	Human	Monopolar RF	Articular cartilage of the knee joint	This in vitro study on human articular cartilage found that RF treatment preserved proteoglycan content but caused collagen denaturation. Monopolar RFE created a smooth surface and reduced catabolic enzyme activity, though at the cost of collagen damage and superficial chondrocyte death

Abbreviations: NRCT, non‐randomised control trial; RCT, randomised control trial; RF, radiofrequency.

### Joint studied

The most frequently studied joint was the knee, reported in the majority of studies across human, ovine, swine, and bovine models. Studies investigating cartilage from the tibial growth plate were also common in swine and bovine models. In contrast, a limited number of studies assessed spinal cartilage and equine articular cartilage.

### Radiofrequency characteristics

Some studies in the literature included the type of RF techniques utilised (i.e., monopolar [13, 27, 28, 31, 33, 35, 54], bipolar [7, 10, 11, 41, 42, 43, 44, 52] or both [6, 11, 31, 49] while others did not add this information in their works [12, 15, 21, 45, 51].

The RF generator used were: Apollo MP50 and Synergy console, both Arthrex, Naples, FL [19], Mitek VAPR [6, 7, 11, 43], Oratec Vulcan EAS [6, 11, 12, 31, 41], ArthroCare 2000 [11, 12, 21, 31, 49], Oratec RF [27], Cool‐tip RF [51], Atlas System (ArthroCare Corporation) [13, 52], Force FX‐C Valley Lab [13, 14], Baylis Medical Company Inc., PMG‐115 TD) [42], ElectroThermal System ORA‐50 [33], Autocon Arthro (Karl Storz GmbH, Tuttlingen, Germany) [23], with the RF wand: Arthrocare Covac 50 [11, 12, 31, 49], Tac‐c probe by Oratec [11, 27, 33], Tac‐c II [11, 12], Paragon T2 wand [13, 21], Saphyre 60° angle bipolar ablation probe [41], Mitek VAPR thermal electric [43], Prototype probe (PPC) [49], Cool‐tip Coviden probe [51], MultiVac TriStar 50 probe (ArthroCare) [52], Razor 2.5 mm ArthroWand high‐frequency electrode (ArthroCare, Sunnyvale, CA) [15] and the hardware used was not addressed in some studies [6, 7, 44, 45]. The generators were set at 10 W [27] 15 W [6, 12, 27, 31, 33, 54], 20 W [43], 25 W [13, 14], 30 W [6, 11], 40 W [43, 44], 60 W [13, 23, 28, 43], 70 W [41], 80 W [49], 90 W [7] and 155–170 W [13] for some studies it wasn't possible to determine the wattage [21, 35, 42, 45, 51, 52].

### Quality assessment of the studies

The selected studies were all checked for biases through ROB2 for RCT and ROBINS‐I [46, 47] for NRCT. It was found that of the 12 NRCTs and two controlled laboratory studies: seven were found to be at a “Low risk of bias” [21, 28, 31, 32, 41, 52, 54], four were at a “Moderate risk of bias” [5, 19, 42, 43] and three were at a “Serious risk of bias” [23, 44, 45]. The quality of the NRCTs included was reported in Figure 2. Of the 13 RCTs it was found that 10 [6, 7, 10, 11, 12, 13, 14, 22, 27, 33, 35] were at “moderate risk of bias”, and three were at “low risk of bias” [35, 49, 52]. No RCTs were found to have a high risk of bias. The quality of the RCTs included was reported in Figure 3.

### Effects on cartilage

Of the 28 studies included in this review, 16 reported varying degrees of damage to the cartilage. In contrast, 12 [5, 13, 22, 27, 28, 35, 41, 43, 44, 49, 52, 54] have reported either no damage or acceptable damage, especially compared with other techniques like MD. Of the five studies that compared MRF with BRF 4 [6, 10, 11, 49] reported more damage with BRF.

Several studies did not report the type of RF used in general reporting results on RF. RF was detrimental compared to MD [12, 15]. The assessment of cartilage integrity was conducted using a range of qualitative and quantitative scoring systems. The most commonly adopted histological scoring methods included the Modified Mankin Score [38], the Osteoarthritis Research Society International (OARSI) Score [37] and the International Cartilage Repair Society (ICRS) Score [50], which were used to quantify the extent of chondrocyte death, matrix degradation, and surface integrity following RF treatment [24].

Of the 28 studies included in this review, 16 reported varying degrees of cartilage damage following RF treatment, while 12 studies [5, 13, 22, 27, 28, 35, 41, 43, 44, 49, 52, 54] described either no damage or an acceptable level of tissue alteration.

MRF was found to cause less thermal damage and lower chondrocyte death rates than BRF in 4 comparative studies [6, 10, 11, 49].

BRF led to greater penetration depth and chondrocyte loss, reaching the subchondral bone in severe cases.

Studies comparing RF to mechanical debridement yielded conflicting results. Some found RF to be more effective in smoothing cartilage surfaces with lower complication rates, while others reported that RF caused higher chondrocyte death and more morphological disruption than mechanical debridement.

Long‐term outcomes remain unclear, as only two studies assessed the persistence of cartilage alterations beyond 24 weeks. These findings highlight the heterogeneity in RF applications and outcomes, suggesting that factors such as probe type, power setting, and exposure time significantly influence tissue effects.

Findings differed between human and animal studies. In human‐derived cartilage specimens, studies consistently reported structural disruption, thermal damage, and chondrocyte death, particularly with BRF, which exhibited deeper thermal penetration compared to monopolar MRF.

Animal studies showed similar trends but with greater variability due to differences in species, cartilage thickness, and experimental settings. Ovine and bovine models exhibited cartilage damage similar to human specimens, with BRF causing deeper thermal penetration and greater chondrocyte apoptosis. Swine and rabbit models, often used for early‐stage studies, revealed increased vulnerability to RF‐induced damage, likely due to thinner cartilage layers and higher metabolic activity.

Regarding follow‐up periods, the majority of studies assessed short‐term outcomes, with only a few studies evaluating the long‐term effects of RFE. Kaab et al. [23] and Lu et al. [33] reported persistent cartilage alterations at 24 weeks post‐treatment, raising concerns about the potential for delayed degeneration.

Overall, while MRF appears to cause less extensive cartilage damage than BRF, significant heterogeneity in study methodologies, power settings, and follow‐up durations makes it difficult to establish definitive clinical recommendations. Future research should focus on standardising assessment methods and evaluating long‐term functional outcomes.

## DISCUSSION

The main finding of this study is that while RF is widely used in cartilage treatment, its effects remain controversial, with MRF generally causing less thermal damage than BRF. Although some studies suggest that RF may offer advantages over mechanical debridement, the evidence is inconsistent, and concerns remain regarding long‐term cartilage viability and safety.

While species‐related differences in cartilage response to RFE are important, the variability in study methodologies, RF settings, and cartilage properties across models makes it more appropriate to structure the discussion based on key outcome parameters rather than by species alone. This approach allows for a more comprehensive comparison of findings while ensuring that species‐specific differences are still acknowledged where relevant.

In the present article, a multitude of surgical techniques were reported. However, there is no consensus on the safest or the most effective. The first differentiation that should be pointed out is between MD and RF. In general, the two techniques both attempt to smooth over fibrillated cartilage. MD works by physically shaving off fibrillated cartilage and, in doing so, smoothing the lesioned area of the cartilage [48]. When this technique is compared with RF by the studies included in this review, the consensus appeared to favour RF over MD [6, 15, 28, 49]. On the other hand, Edwards et al. [12] reported that RF causes more chondrocyte death and loss of morphology than MD. As described above, all the studies included in this review report variable amount of damage to cartilage when using RF with only a minority reporting negligible damage [27, 44, 54]. Even among the types of RF the damage reported appears to be dependent on the kind of RF used as there is a clear tendency towards monopolar RF being a safer option when compared to bipolar RF, as studies generally show that it causes less thermal damage to cartilage as reported by multiple studies [6, 10, 11, 28] as a testament to this it has been found out that BRF causes more profound chondrocyte death as deep as the subchondral bone as reported by Yasura et al. [54]. In addition, monopolar RF was found to cause minor damage to the cartilage, as written by Lopez et al. [27].

While there is substantial evidence about the effects of MRF, only two studies reported on the long‐term effects of this kind of treatment. Kaab et al. [23] report damage to the cartilage after 24 weeks, and Lu et al. [33] report damage in the long term. The only other study reporting the effects of RF on cartilage fails to specify the kind of RF used by Koller et al. [21], this lack of literature warrants further research on the topic.

Moreover, several variables seem to play a role in the amount of damage sustained by the cartilage. First, Lu et al. [31] report that the depth of chondrocyte death is directly proportional to the time. Similar results were obtained by Peng et al. [41].

Lu et al. [29] reports that a flowing fluid is preferable to avoid heating the joint liquid above the threshold of 45° [29], at which chondrocytes start to die. This finding is shared by McCormick et al. [34] which advocates for an intermittent 5 s puled lavage to maintain the temperature under 50°C and Zoric et al. [56] who tested temperatures with different irrigation modalities. The authors reported that even a short interruption in irrigation could lead to an elevation in temperature.

Moreover, the power setting appears to correlate to the damage amount. According to Mitchell et al. [35], a lower power setting generally leads to less cartilage damage. Wang et al. [52] reported that the high‐power ablation mode causes less thermal damage than the less powerful coagulation mode, so there is no true consensus. Furthermore, exposure time to RF also appears to influence cartilage damage, as Lu et al. [31] and Peng et al. [41] reported. The authors described a positive correlation between exposure time to RF and damage to the cartilage.

The cutting with RFs is performed by emission of continuous radio frequency waveforms, which will ablate soft tissue leaving a collateral damage zone. Two basic RF systems with controlled probes for clinical application, monopolar and bipolar, can accomplish this process. The former works by sending a current through the region that needs to be ablated; this current heats the tissue due to its higher resistance. The current then moves through the patient to grounding pads, usually applied to the patient's skin with the inherent risk of skin pad burn [16]. In contrast, BRF works by sending a current as a monopolar does but then converging this to a second probe and avoiding the need for pads altogether [16]. As previously seen, this technique has faults, as exposure to higher RF leads to a higher risk of chondrocyte death as the threshold for tissue thermal damage at 50°C is 2 s. The apparent advantage of BRF is that the plasma layer can reduce the permeability of the cartilage by annealing it. This has the inherent consequence of avoiding cartilage enzyme infiltration, which at face value appears beneficial. Still, increased MMP‐13 tissue immunoreactivity and collagen loss could prove to be detrimental in the long term [3, 6, 49]. While this systematic review focuses on preclinical studies, some clinical investigations have explored RF applications in joint pathology. For instance, RF ablation has been used for pain management in osteoarthritis, but these studies do not evaluate direct cartilage effects [18, 55]. Similarly, recent studies on ultrasound‐guided RF ablation for synovial hyperplasia suggest a potential role in inflammatory joint diseases [53]. However, no high‐quality clinical trials have specifically assessed the long‐term safety and efficacy of RF treatment for cartilage lesions, highlighting a critical gap in translational research.

Based on preclinical data, RF has been proposed as a treatment for articular cartilage defects, particularly in cases of mild‐to‐moderate chondral damage where minimally invasive smoothing of fibrillated cartilage is desired. Some authors suggest that MRF may serve as an adjunct to arthroscopic debridement, reducing irregularities while minimising chondrocyte loss [6, 10, 28]. However, concerns regarding long‐term cartilage integrity remain unresolved. Clinical indications may also extend to post‐traumatic chondral lesions, but further studies are needed to define optimal power settings, application time, and patient selection criteria.

### Limitations

One of the main limitations of this systematic review is the heterogeneity of study designs, despite the presence of 12 RCTs. These trials vary significantly in terms of experimental models, power settings, treatment protocols, and evaluation methods, making it difficult to derive definitive clinical recommendations. Moreover, most studies focus on short‐term outcomes, with very few assessing long‐term cartilage viability beyond several months. The lack of standardised outcome measures further limits the ability to compare results across studies. A meta‐analysis could not be conducted due to differences between the specimens assessed and the technique adopted (monopolar, bipolar, type of fluid, etc.). Lastly, only articles in English were considered, restricting the pool of available literature. Future research should focus on high‐quality, well‐controlled trials with longitudinal follow‐up to better understand the long‐term safety and efficacy of RF treatment for cartilage lesions.

## CONCLUSION

This systematic review highlights the current evidence on the effects of RF on cartilage. Although RF technology has been widely used in cartilage treatment, the available preclinical studies indicate a lack of consensus on its long‐term safety and efficacy. MRF appears to cause less thermal damage than bipolar RF, but conflicting results exist regarding its superiority over mechanical debridement. The overall risk‐benefit profile of RF remains unclear due to significant variability in study methodologies, power settings, and assessment criteria. Therefore, further high‐quality research, including well‐designed clinical trials, is needed to establish clear guidelines on the use of RF for cartilage treatment and to fully understand its potential risks and benefits.

## AUTHOR CONTRIBUTIONS

*Conceptualisation*: Umile Giuseppe Longo and Sergio De Salvatore. *Methodology*: Daniela Lo Presti. *Software*: Alessandro de Sire and Dario Neyjat. *Validation*: Umile Giuseppe Longo and Kristian Samuelsson. *Formal analysis*: Youssef Raffoul Arab Orozco. *Investigation*: Sergio De Salvatore. *Resources*: Francesca De Tommasi. *Data curation*: Margaux D'Hooghe. *Writing—original draft preparation*: Dario Neyjat and Youssef Raffoul Arab Orozco. *Writing—review and editing*: Sergio De Salvatore. *Visualisation*: Umile Giuseppe Longo. *Supervision*: Umile Giuseppe Longo. *Project administration*: Kristian Samuelsson. All authors have read and agreed to the published version of the manuscript.

## CONFLICT OF INTEREST STATEMENT

K.S. is a member of board of directors of Getinge AB (publ) and medtech advisor to Carl Bennet AB.

## ETHICS STATEMENT

The authors have nothing to report.

## Data Availability

The data that supports the findings of this study are available in the supplementary material of this article.
